# Water column dynamics control nitrite-dependent anaerobic methane oxidation by *Candidatus* “Methylomirabilis” in stratified lake basins

**DOI:** 10.1038/s41396-023-01382-4

**Published:** 2023-02-20

**Authors:** Guangyi Su, Moritz F. Lehmann, Jana Tischer, Yuki Weber, Fabio Lepori, Jean-Claude Walser, Helge Niemann, Jakob Zopfi

**Affiliations:** 1 Department of Environmental Sciences, University of Basel, Basel, Switzerland; 2 State Key Laboratory of Marine Environmental Science, College of Ocean and Earth Sciences, Xiamen University, Xiamen, China; 3 Department for Environment, Constructions and Design, University of Applied Sciences and Arts of Southern Switzerland (SUPSI), Mendrisio, Switzerland; 4 Genetic Diversity Centre (GDC), ETH Zürich, Zürich, Switzerland; 5 Department of Marine Microbiology and Biogeochemistry, NIOZ Royal Institute for Sea Research and Utrecht University, Texel, The Netherlands

**Keywords:** Biogeochemistry, Water microbiology, Limnology, Microbial ecology

## Abstract

We investigated microbial methane oxidation in the water column of two connected but hydrodynamically contrasting basins of Lake Lugano, Switzerland. Both basins accumulate large amounts of methane in the water column below their chemoclines, but methane oxidation efficiently prevents methane from reaching surface waters. Here we show that in the meromictic North Basin water column, a substantial fraction of methane was eliminated through anaerobic methane oxidation (AOM) coupled to nitrite reduction by *Candidatus* Methylomirabilis. Incubations with ^14^CH_4_ and concentrated biomass from this basin showed enhanced AOM rates with nitrate (+62%) and nitrite (+43%). In the more dynamic South Basin, however, aerobic methanotrophs prevailed, *Ca*. Methylomirabilis was absent in the anoxic water column, and no evidence was found for nitrite-dependent AOM. Here, the duration of seasonal stratification and anoxia seems to be too short, relative to the slow growth rate of *Ca*. Methylomirabilis, to allow for the establishment of anaerobic methanotrophs, in spite of favorable hydrochemical conditions. Using 16 S rRNA gene sequence data covering nearly ten years of community dynamics, we show that *Ca*. Methylomirabilis was a permanent element of the pelagic methane filter in the North Basin, which proliferated during periods of stable water column conditions and became the dominant methanotroph in the system. Conversely, more dynamic water column conditions led to a decline of *Ca*. Methylomirabilis and induced blooms of the faster-growing aerobic methanotrophs *Methylobacter* and *Crenothrix*. Our data highlight that physical (mixing) processes and ecosystem stability are key drivers controlling the community composition of aerobic and anaerobic methanotrophs.

## Introduction

Lakes are important sources of methane (CH_4_), a potent greenhouse gas in the atmosphere [1]. A large fraction of methane is produced by anaerobic methanogenic archaea in lake sediments, from where it may escape by ebullition or diffusion into deep waters. Many studies have evidenced aerobic methane oxidation at the sediment surface and in the oxic water column of lakes [2–7]. Within sediments or anoxic bottom waters, methane may be oxidized anaerobically. At least in the marine realm, this anaerobic oxidation of methane (AOM) substantially reduces methane emissions to the atmosphere [8], and is mainly performed by microbial consortia of anaerobic methanotrophic archaea (ANME-1, -2, and -3) and sulfate-reducing bacteria (SRB) [9–12]. Recent studies have reported other electron acceptors of potential importance for AOM, including nitrogenous compounds [13**–**15], iron and/or manganese oxides [16**–**19], and possibly humic substances [20, 21].

Especially in freshwater environments, AOM with electron acceptors other than sulfate may represent a significant methane sink [17, 22–25]. For nitrogen-dependent anaerobic oxidation of methane (N-AOM), two different modes have been identified. Firstly, the bacterial oxidation of methane with nitrite by e.g., *Candidatus* Methylomirabilis oxyfera [26**–**28], where oxygen is produced by intracellular disproportionation of nitric oxide to nitrogen and oxygen, which is then used for intra-aerobic methane oxidation [14]. Secondly, true anaerobic oxidation of methane coupled to nitrate reduction, catalyzed by methanotrophic archaea (e.g., *Candidatus* Methanoperedens nitroreducens) [15]. Although the exact metabolic mechanisms of nitrate/nitrite-dependent AOM are not entirely clear, this process has been observed in freshwater environments [26, 29–35] and in marine oxygen minimum zones [36].

Given the prevalence of nitrate in freshwater lakes, N-AOM may play an important role in the reduction of methane emissions. In lacustrine environments, highest methane oxidation rates were often observed near oxic/anoxic transition zones at the sediment-water interface [2, 37–40] or in the water column of stratified lakes [3, 41]. However, methane consumption at these boundaries was generally thought to be carried out by (micro-)aerobic methanotrophs, fueled by oxygen supplied by diffusion, intrusion events, or cryptic production [2, 5, 6, 42–44]. Redox transition zones also represent sites where nitrate/nitrite is typically regenerated through the oxidation of ammonium and reduction of nitrate by nitrogen-transforming microorganisms [45**–**47]. Hence, there, N-AOM might be masked by, or misinterpreted as, aerobic methane oxidation [48]. As a result, methane oxidation with nitrate/nitrite as terminal electron acceptors may play a greatly underappreciated role in lakes.

Lake Lugano (Switzerland) provides excellent conditions for studying physico-chemical controls on AOM as a function of ecosystem dynamics, as the lake comprises two interconnected basins that differ significantly in terms of mixing regime, water column stability, and hence redox conditions. We quantified methane oxidation rates in the water column of the two basins, performed incubation experiments to determine the effectiveness of different electron acceptors for AOM, and applied 16 S rRNA gene amplicon sequencing to identify key taxa of aerobic and anaerobic methanotrophs. Archived DNA samples from the North Basin allowed us to track the multiannual dynamics of *Ca*. Methylomirabilis and other methanotrophs in the years following an exceptional mixing event in 2005 and 2006.

## Materials and methods

### Site description and sampling

Lake Lugano is located at the Swiss-Italian border and consists of two hydrodynamically contrasting basins that are separated by a causeway, which was built on a natural moraine. The eutrophic 95 m-deep South Basin undergoes seasonal stratification with the development of a benthic bacterial nepheloid layer and anoxia during summer and fall [49]. The mesotrophic 288 m-deep North Basin is nearly permanently stratified since the 1960’s, and a chemocline at about 100–130 m separates the oxic mixed layer (mixolimnion) from the anoxic part of the water column (monimolimnion) [49]. Only in 2005 and 2006, stratification was interrupted by exceptional mixing of the whole water column at the end of cold and windy winters, causing transient oxygenation of the monimolimnion [50, 51].

Water samples were collected in late November 2016, near the deepest points of the South Basin (45°57′N, 8°54′E) and the North Basin (46°00′N, 9°01′E). Oxygen concentrations were measured using a conductivity, temperature, and depth (CTD) probe (Idronaut Ocean Seven 316 Plus). Water samples from distinct depths were collected using 5L-Niskin bottles. Subsamples were taken directly from a Niskin bottle and filtered (0.45 µm) and/or processed as outlined below. Water samples for methane oxidation measurements were collected into 20 mL glass vials, allowing water to overflow for about 2–3 volumes. They were filled completely, and care was taken not to introduce any air bubbles. The vials were crimp-sealed with Br-butyl rubber stoppers [52]. Samples for methane concentration measurements were collected in 120 mL serum bottles, crimp-sealed with thick butyl rubber stoppers and a 20 mL air headspace was created before fixing the sample by adding 5 mL of 12.5 M NaOH.

### Analytical methods

Methane concentrations in the headspace of NaOH-fixed water samples were measured using a gas chromatograph (SRI 8610C, SRI Instruments) with a flame ionization detector [49]. Ammonium (NH_4_^+^) concentrations were determined colorimetrically using the indophenol reaction, and nitrite (NO_2_^–^) using Griess reagent [53]. NO_x_ (nitrate plus nitrite) was determined by chemo-luminescence detection using a NO_x_-Analyzer (Antek Model 745). Nitrate (NO_3_^−^) concentrations were calculated from the difference between NO_x_ and NO_2_^–^. Filtered samples for sulfide concentrations were fixed with zinc acetate immediately after sampling and analyzed photometrically in the laboratory [54]. Sulfate was analyzed by ion chromatography (940 Professional IC Vario, Metrohm, Switzerland). Water samples for dissolved iron (Fe^2+^) and manganese (Mn^2+^) were fixed with HCl (0.5 M final conc.) after filtration through a 0.45 µm membrane filter, and analyzed using inductively coupled plasma optical emission spectrometry (ICP-OES). Total Fe and Mn concentrations were determined in fixed unfiltered water samples. Concentrations of Fe^2+^ were additionally determined photometrically using the ferrozine assay [55]. Particulate iron was calculated from the difference between the total Fe, and the dissolved Fe^2+^ in the filtered sample.

### Methane oxidation rate measurements

Potential methane oxidation rate (MOR) profiles in the water column were determined using short-term incubations with trace amounts of tritium-labeled methane (^3^H-CH_4_) [56]. Tritiated methane has a higher specific activity than ^14^C-CH_4_; thus, less tracer is required for MOR determination, and the rates are less affected by artificially increased CH_4_ concentrations. Upon the retrieval of water samples from different depths, 5 µL anoxic ^3^H-CH_4_ solution (~1.8 kBq) was injected into the 20 mL bubble-free glass vials, and samples were incubated in the dark at 4 °C for 42 h, as described earlier [56]. Depending on the original oxygen content in the water sample, measured MOR represents aerobic and/or anaerobic methane oxidation rates.

### Anoxic incubation experiments with ^14^C-labeled methane

Long-term laboratory incubation experiments were set up with microbial biomass from anoxic water layers of both basins to test different electron acceptors for their potential to stimulate anaerobic oxidation of methane (AOM). Briefly, the biomass of a 500 mL water sample was collected on a glass fiber filter, which was then transferred to a 120 mL serum bottle containing 100 mL anoxic artificial lake water. The bottles were subsequently purged with oxygen-free nitrogen until the oxygen concentrations in the control bottles, equipped with trace oxygen sensor spots (TROXSP5, Pyroscience, Germany), were below the detection limit (0.1 µM). Under nitrogen-atmosphere in an anoxic chamber (GS Glovebox Systemtechnik, Germany), potential electron acceptors were added from anoxic stock solutions to a final concentration of 4 mM (nitrate, nitrite) and 2 mM (sulfate), respectively. Molybdate, a specific inhibitor of dissimilatory sulfate reduction [57], was added to some incubations at 4 mM final concentration to test for sulfate-dependent anaerobic methane oxidation. After these additions, the bottles were filled headspace-free with anoxic medium [25], and closed with gray butyl rubber stoppers. To remove the oxygen dissolved in the elastomer, the stoppers were boiled in water, and afterward stored under helium gas until use. Finally, 10 µL of ^14^CH_4_ tracer solution (~2.4 kBq) was injected, and samples were incubated at 25 °C for 16 days and 32 days. To exclude potential oxygen contamination during the long incubation time, the closed incubation bottles were kept permanently under nitrogen-atmosphere in an anoxic chamber. Both living controls (without added electron acceptors) and NaOH-killed controls (pH > 13) were treated in the same way, and incubated in parallel for the two basins. At the end of the incubation, a 20 mL nitrogen headspace was created. Biological activity was stopped by adding 5 mL 12.5 M NaOH solution (pH > 13). The radioactivity of residual ^14^CH_4_ (combusted to ^14^CO_2_), ^14^CO_2_ produced by methane oxidation, and radioactivity in the remaining samples were determined by liquid scintillation counting [58, 59].

### DNA extraction, PCR amplification, sequencing, and data analysis

Water samples from different depths of the two basins were collected and sterile-filtered for biomass, using 0.2 µm polycarbonate membrane filters (Cyclopore, Whatman). Biomass DNA was then extracted using FastDNA SPIN Kit for Soil (MP Biomedicals) following the manufacturer’s instructions. A two-step PCR approach [60] was applied in order to prepare the library for sequencing at the Genomics Facility Basel. Briefly, 10 ng of extracted DNA were used for a first PCR using the primers 515F-Y (5′-GTGYCAGCMGCCGCGGTAA) and 926 R (5′-CCGYCAATTYMTTTRAGTTT-3’) targeting the V4 and V5 regions of the 16 S rRNA gene [61], with good and comparable coverage for Bacteria (84.5%), Archaea (81.05%), and Eukaryota (80.8%) (https://www.arb-silva.de/search/testprime/). The primers of the first PCR were composed of the target region and an Illumina Nextera XT-specific adapter sequence. Four sets of forward and reverse primers, which contained 0–3 additional and ambiguous bases after adapter sequence, were used in order to introduce frame shifts to increase complexity (electronic supporting material, Table S1). Sample indices and Illumina adapters were added in a second PCR of 8 cycles. Purified, indexed amplicons were finally pooled at equimolar concentration, denatured, spiked with 10% PhiX, and sequenced on an MiSeq platform (Illumina) using the 2 × 300 bp paired-end protocol (V3-Kit). The quality of the raw reads was checked using FastQC (v 1.2.11; Babraham Bioinformatics). FLASH [62] was used to merge forward and reverse reads into amplicons of about 374 bp length with an average merging rate of 96%, allowing a minimum overlap of 15 nucleotides and a mismatch density of 0.25. Full-length primer regions were trimmed using USEARCH (v10.0.240), allowing a maximum of one mismatch. The merged and primer-trimmed amplicons were quality-filtered (size range: 250–550, no Ns, minimum average quality score of 20) using PRINSEQ [63, 64, 65]. Amplicon Sequence Variants (ASVs) were identified by denoising amplicons to zero radius OTUs (ZOTUs) using the UNOISE algorithm in USEARCH. Taxonomic assignment was done with SINTAX (v11.0.667_i86linux64, Edgar, 2016) and the SILVA 16 S rRNA gene reference database v138 [66]. Downstream sequence analysis was done in R v3.5.1 using “phyloseq” v1.25.2 [67], “vegan” [68], and “ggplot2” [69], as outlined in the electronic supporting material. A total of 57 North Basin water column samples from 2015 to 2018 were used to perform the network analysis. After filtering out ASVs with mean relative abundances of <0.1%, a total of 100 ASVs remained for network construction, using pairwise Pearson correlation. The correlation matrix was converted into an adjacency matrix using a threshold of ≥0.75. The network was constructed using the “igraph” package in R, and visualized using “tidygraph”, and “ggraph”. The abundance of *Ca*. Methylomirabilis was determined by qPCR according to Ettwig et al. [26].

## Results and discussion

### Hydrochemistry and methane oxidation rates

**Fig. 1:**
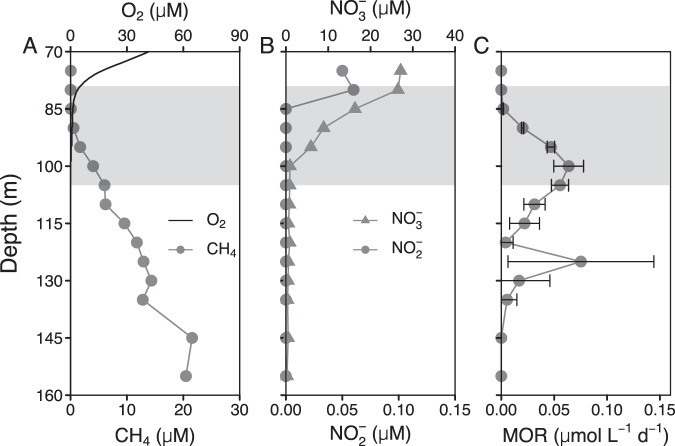
Water column profiles in the North Basin of Lake Lugano (November 2016). (**A**) oxygen (O_2_) and methane (CH_4_) concentrations, (**B**) nitrate (NO_3_^–^) and nitrite (NO_2_^–^) concentrations and (**C**) methane oxidation rates (MOR). The gray area represents the redox transition zone, starting at O_2_ < 5 µM, and reaching to the depth where sulfide concentrations start to rise above background levels (see also Supplementary Fig. S3). Error bars of MOR represent standard deviations (*n* = 3).

The water column of the deep North Basin is considered meromictic (i.e., permanently stratified). At the time of sampling for methane oxidation rate measurements in November 2016, the redox transition zone extended from 79 to 105 m depth; as defined here, with an upper boundary set at O_2_ < 5 µM, and a lower boundary set where oxygen sensitive reduced chemical species like Fe^2+^ or H_2_S start to rise above background levels (Fig. 1A, Supplementary Fig. S3). Methane concentrations increased from very low levels (<0.1 µM) at the upper redox transition zone to 20 µM at 155 m. At this depth, nitrite was below the limit of detection (0.02 µM) and nitrate in the sub-micromolar range (<1 µM, Fig. 1B).

Vertical profiles of potential rates of methane oxidation (MOR) in the North Basin showed a bimodal pattern (Fig. 1C). The upper peak of methane oxidation rates (0.06 ± 0.01 µmol L^−1^ d^−1^) at 100 m was at least partly due to the activity of aerobic methanotrophs, thriving under microoxic conditions, as reported before [3]. However, methane oxidation continued below the redox transition zone, and a secondary methane oxidation rate maximum of 0.08 ± 0.07 µmol L^−1^ d^−1^ was detected at 125 m. A similar bimodal methane oxidation rate distribution has been observed in the North Basin water column before, whereby, at the time, both the oxycline and the two separate methane oxidation rate maxima were located at greater depths [3].

**Fig. 2:**
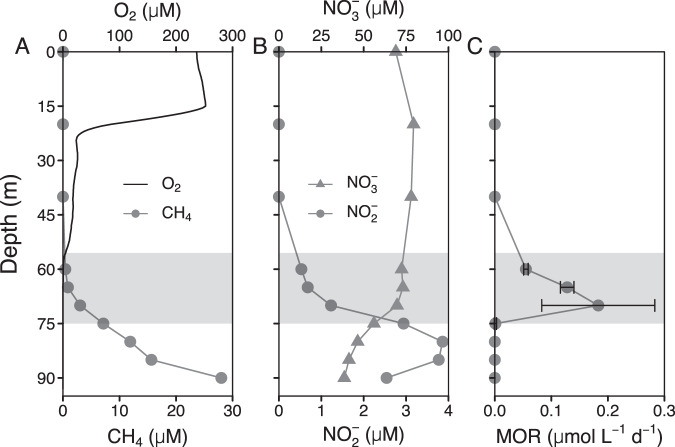
Water column profiles in the South Basin of Lake Lugano (November 2016). (**A**) oxygen (O_2_) and methane (CH_4_) concentrations, (**B**) nitrate (NO_3_^–^) and nitrite (NO_2_^–^) concentrations, and (**C**) methane oxidation rates (MOR). The gray area represents the redox transition zone, starting at O_2_ < 5 µM, and reaching to the depth where Fe^2+^ rises above background concentrations (see also Supplementary Fig. S3). Error bars of MOR represent standard deviations (*n* = 3).

In the eutrophic South Basin, seasonal near-bottom anoxia typically starts to develop in early summer, and is associated with elevated turbidity in the deep waters. This benthic nepheloid layer extends from the lake ground up to the oxic/anoxic interface and consists of microbial biomass, produced locally and to large parts by methanotrophs [4]. Its development starts at the sediment-water interface and then progressively expands 10–20 m into the water column, following the rising redox transition zone [4]. During the time of sampling, in November 2016, the redox transition zone extended from 55.5–75 m (Fig. 2A and Supplementary Fig. S4), below which, in contrast to the North Basin, considerable amounts of nitrate (38–73 µM) and nitrite (1.2–3.9 µM) were present (Fig. 2B). Methane concentrations below the redox transition zone increased towards the sediment and reached levels that were comparable (28 µM) to those in the North Basin. In the South Basin, a single peak of methane oxidation rate (0.18 ± 0.1 µmol L^−1^ d^−1^) was observed at 70 m depth, towards the lower boundary of the redox transition zone (Fig. 2C). Although Type I methane-oxidizing bacteria (MOB) were shown previously to dominate the biomass in the benthic nepheloid layer, where the highest methanotrophic activity was observed, it remained unclear whether the observed activity was soley due to aerobic methanotrophs [4]. Particularly the presence of both nitrate/nitrite, but also sulfate, Fe(III)- and Mn(IV)oxides (Supplementary Fig. S4) within the benthic nepheloid layer bears the potential that methane could be oxidized anaerobically with either of these oxidants.

Methane oxidation within oxic/anoxic transition zones of other stratified lakes has often been attributed to aerobic methanotrophs [6, 70]. In lakes with shallow redox transition zones, cryptic oxygen production by phototrophs could sustain aerobic methane oxidation even in seemingly anoxic waters [5, 71]. At the depths of the redox transition zone in Lake Lugano, particularly in the North Basin, oxygen production by phototrophs is an unlikely mechanism. Alternatively, Blees et al. [3] suggested that aerobic methane oxidizers below the redox transition zone can survive prolonged periods of oxygen starvation, and can resume high methane oxidation activity upon episodic downwelling of oxygen, for example during cooling events. Yet potential mechanisms that inject oxygen to the deep hypolimnion were not investigated, and it remained speculative if, and to which depth, such events may occur. Thus, methane oxidation below the redoxcline in the Lake Lugano North Basin may indeed be anaerobic.

### Evidence for nitrate/nitrite-dependent AOM

To test for the presence of active anaerobic methanotrophs, and to indentify potential oxidants for methane, we set up anoxic incubation experiments with ^14^CH_4_ as substrate, different electron acceptors (nitrate, nitrite, sulfate), and concentrated biomass. The biomass was collected from 85–90 m in the South Basin, a depth well below the redox transition zone at this time of sampling, but where nitrate, nitrite, and sulfate were present. Biomass from the North Basin was collected at 105–110 m, where nitrite was undetectable but low levels of nitrate and sulfate were still present.

**Fig. 3:**
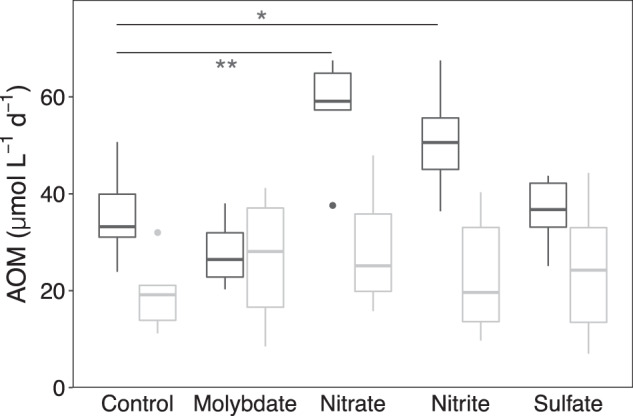
Effect of different electron acceptors on AOM rates in comparison to control experiments without addition of electron acceptors (*n* = 6). The incubations were conducted with concentrated biomass collected in November 2016 from anoxic water layers in both North Basin (black boxplots) and South Basin (gray boxplots), and amended with ^14^CH_4_ and different oxidants (nitrate, nitrite, sulfate) or molybdate as inhibitor of sulfate reduction. In the killed controls (*n* = 3; not shown) no tracer conversion was observed after 32 days.

When biomass from the meromictic North Basin was used, we found that both nitrate (*p* < 0.01) and nitrite (*p* = 0.05) stimulated AOM rates significantly (Fig. 3, Table S4). Compared to the controls without additions, AOM rates increased by an average of 62% and 43% in the presence of nitrate (57.8 ± 10.8 µmol L^−1^ d^−1^) and nitrite (50.9 ± 10.8 µmol L^−1^ d^−1^), respectively. There was no significant difference between the controls and amendments with sulfate (Table S4B).

In the South Basin, no significant stimulation of methane oxidation occurred with any of the added electron acceptors (Fig. 3 and Table S4), suggesting that these are not immediate oxidants for methane, and AOM was not a major mode of methane removal in spite of the presence of nitrate, nitrite, and sulfate. However, the methane oxidation rates in the South Basin incubations increased with a longer incubation time (i.e., 32 days, data not shown), independently of the added compounds, including molybdate. The observed stimulation of AOM thus seems independent of sulfate-reducing bacteria. In turn, the question arises as to what was the electron acceptor supporting AOM under these conditions, and more generally, what was the oxidant for methane in the unamended controls? Non-zero AOM rates in controls are frequent [3, 72, 73], possibly resulting from minor oxygen contamination at the start of the experiment or during sampling. Although greatest care was taken to prevent oxygen contamination during preparation, incubation, and sampling, by executing all steps in a nitrogen-flushed anoxic chamber, we did not add a reducing agent to chemically remove any traces of oxygen. Thus, if trace amounts of oxygen were still present in the incubations, they might have served as substrate for the methane monooxygenase. After the oxygen-dependent initial hydroxylation of methane to methanol, its further transformation could proceed anaerobically by fermentation, whereby hydrogen, formate, acetate, and other compounds are produced [74]. While trace oxygen contamination may be the reason for non-zero AOM rates in the living controls, they can not explain the increase in AOM rates between 16 days and 32 days of incubation with South Basin biomass. A possibility, inferred from these incubation experiments, is that the methanotrophs oxidized methane with Fe(III)- or Mn(IV)-oxides [16, 18, 19], which were present in the South Basin (Supplementary Fig. S4). Growing experimental and metagenomic evidence suggests that fermentation and the potential for extracellular electron transfer (e.g., to Fe(III)- or Mn(IV)-oxides) are widespread among freshwater MOB [75, 76]. Particulate metal oxides would accumulate on the filters used to concentrate MOB biomass for the incubation experiments. Close spatial arrangement or even direct contact between methanotrophs and insoluble metal oxides on the filters may have stimulated AOM rates with time. Whereas this hypothesis still needs to be tested for Lake Lugano, incubation experiments with biomass from the North Basin clearly showed that both nitrate and nitrite enhanced methane oxidation under anoxic conditions, providing evidence for active N-AOM in the meromictic North Basin.

### Abundance and diversity of methanotrophic bacteria

Methanotrophy is an important biogeochemical process at the redox transition zones of both lake basins. Up to 32.2% of the 16 S rRNA gene amplicons in the benthic nepheloid layer of the South Basin, and 11.2% at 95 m in the North Basin were related to methanotrophs (Supplementary Excel Table S1). Among the 41 identified ASVs of putative methanotrophs, gamma-proteobacterial type I MOB (31 ASVs) were by far the most important group in terms of relative abundance and diversity. Also, eight ASVs of alpha-proteobacterial type II MOB were detected, but they were generally low in abundance. Furthermore, two identified ASVs (ASV9 and ASV7279) were related to *Ca*. Methylomirabilis (Supplementary Excel Table S1), capable of mediating AOM with nitrite as oxidant [14]. No sequences of anaerobic methane-oxidizing Archaea, such as *Ca*. Methanoperedens, or representatives of the ANME-1, -2 or -3 groups, were detected in any of the samples.

**Fig. 4:**
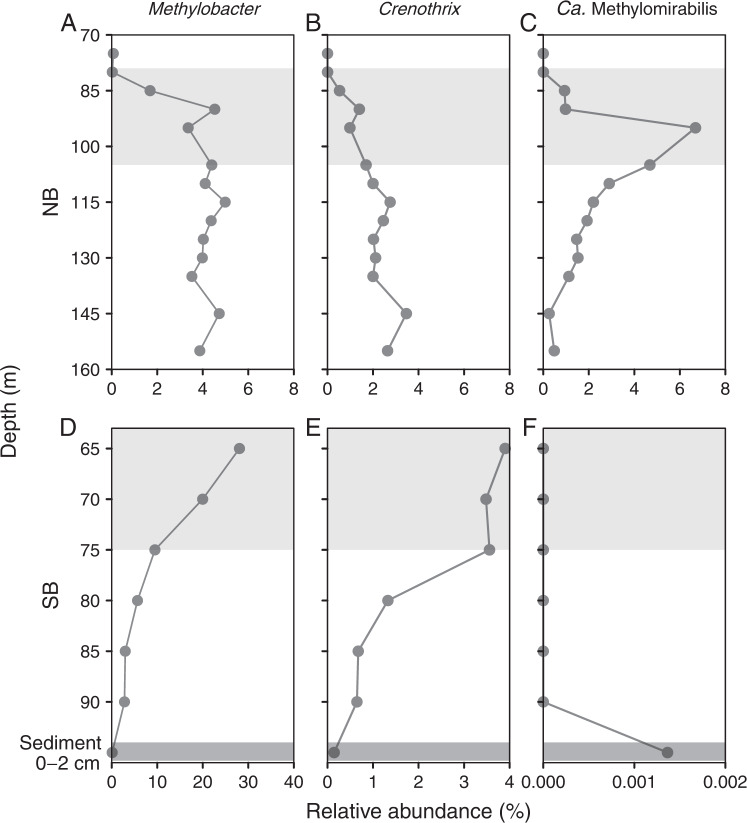
Depth distribution of the main methanotrophs in the water column of Lake Lugano (November 2016). Relative abundances of *Methylobacter*, *Crenothrix*, and *Ca.* Methylomirabilis in the North Basin (NB: **A**–**C**) and South Basin (SB: **D**–**F**). Data are presented as relative read abundances (in %) of 16 S rRNA gene amplicons. The redox transition zone is represented by the light-gray shaded area.

The guild of methanotrophs in the lake was dominated by only seven highly abundant ASVs, with >1% relative abundance in at least one sample, including uncultured representatives of *Methylobacter* sp (ASV5, ASV18, ASV19, ASV42), *Crenothrix* sp. (ASV10, ASV91), as well as *Ca*. Methylomirabilis (ASV9). These seven taxa combined represented >96% of all sequence reads of methanotrophs (Supplementary Fig. S5, Supplementary Excel Table S1). All but *Ca*. Methylomirabilis were present in both the North Basin and the South Basin water column, coexisting, at varying proportions, respectively, in microoxic as well as in anoxic water layers (Fig. 4). *Ca*. Methylomirabilis, however, showed a clear habitat preference for the meromictic North Basin, where ^14^CH_4_ incubations indicated active N-AOM, and where *Ca*. Methylomirabilis was with 6.5% at 95 m the most abundant MOB in November 2016 (Fig. 4).

**Fig. 5:**
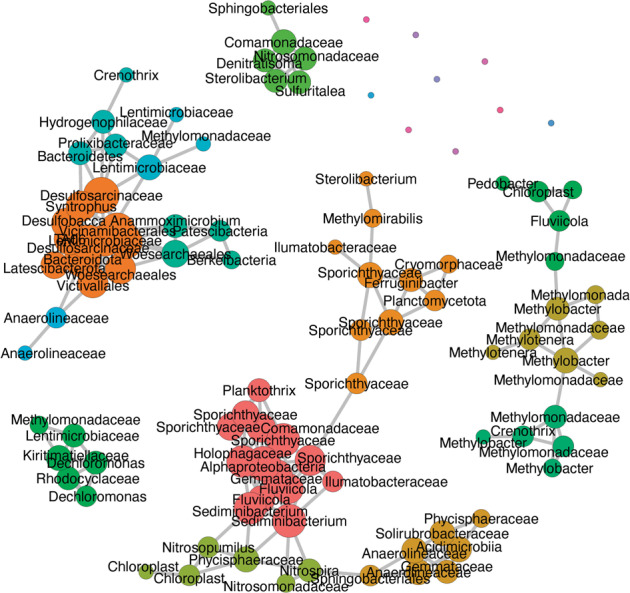
Pearson correlation-based network analysis of water column microbial communities in 57 North Basin water column samples from 2015 to 2018. After filtering out ASVs with mean relative abundances of <0.1%, a total of 100 ASVs were retained for network construction. Nodes of the same color within a cluster indicate that these taxa are interconnected, and the node size indicates the centrality degree.

All three MOB groups, *Methylobacter* sp, *Crenothrix* sp., and *Ca*. Methylomirabilis coexisted in the North Basin water column, where methane consumption rates were highest (Fig. 1, Supplementary Figs. S5 and S6), suggesting that both aerobic and anaerobic methanotrophs work in concert under the microoxic to anoxic conditions in the redox transition zone, and form an efficient pelagic methane filter (Fig. 1). The methane oxidation rate maximum coincided with the maximum abundance of *Ca*. Methylomirabilis, but nitrite, the prime electron acceptor for this organism was undetectable below 85 m depth at the time of sampling. Nitrite could be supplied by ammonia oxidizing archaea or bacteria (Supplementary Fig. S7), or, alternatively, by nitrate-reducing bacteria, as has been proposed previously to meet the nitrite demand of anammox bacteria [47]. In support of the second hypothesis, we find, by correlation-based network analysis, that *Ca*. Methylomirabilis forms a subnetwork together with some nitrate-reducing bacteria, including the denitrifying bacterium *Sterolibacterium* sp. (Fig. 5). In contrast, the typical nitrifiers are associated with, probably degrading, algal biomass. The other main methanotrophs, *Crenothrix* sp. and *Methylobacter* sp., form a separate subnet together with *Methylotenera* sp., suggesting a potential syntrophic interaction of methano- and methylotrophs as recently shown experimentally by ^13^CH_4_ based DNA-SIP [46].

In the eutrophic, seasonally stratified South Basin, 16 S rRNA gene sequences of *Ca*. Methylomirabilis were not detected in the water column, although the chemical conditions seemed favorable for N-AOM, with nitrate and nitrite concentrations reaching 73 µM and 3.9 µM, respectively (Fig. 2). The two lake basins are hydrologically and microbiologically connected, and *Ca*. Methylomirabilis was present in South Basin surface sediments (Fig. 4C). The absence of *Ca*. Methylomirabilis in the South Basin water column is thus not the consequence of limited dispersion, or unsuitable chemical conditions. Rather, it is the limited period of time, during which these chemical conditions prevail, which prevents *Ca*. Methylomirabilis—and other slow growing organisms such as anammox bacteria [47]—from forming a stable and sizeable population in this basin. Indeed, the anoxic stratification period of ~5 months is short compared to an apparent doubling time of 104 days estimated for *Ca*. Methylomirabilis in the North Basin (Supplementary Fig. S8), and faster growing, and potentially metabolically more versatile taxa outcompete *Ca*. Methylomirabilis. In accordance with Blees et al. [4], we find that the assemblage of methanotrophs at the depth interval of maximum methane oxidation (65–75 m) was composed of *Methylobacter* sp. (e.g., ASV5, 14.5% and ASV18, 4.0%) followed by *Crenothrix* sp. (e.g., ASV10, 2.8%) (Fig. 4B and Supplementary Excel Table S1). This latter ASV10 is highly similar to *Crenothrix* sp. D3, which has been shown to contribute to methane oxidation in two other stratified lakes in Switzerland [77]. Despite their occurrence in anoxic waters, these gamma-proteobacterial MOB are still considered aerobic, as they use molecular oxygen for the particulate methane monooxygenase and the initial activation of methane. The apparent absence of true anaerobic methane oxidizers in the South Basin water column is consistent with the lack of significant stimulation of AOM with nitrate/nitrite in the incubations with biomass from this basin (Fig. 3 and Table S4). Nonetheless, genomes of several aerobic methanotrophs, including *Crenothrix*, encode putative nitrate (*narG*, *napA*), nitrite (*nirS*, *nirK*), and/or nitrogen oxide reductases (*norB*) [72, 75, 77, 78]. *Methylomonas denitrificans*, for example, can couple the oxidation of methane (and methanol) to the reduction of nitrate to nitrous oxide under severe oxygen limitation [78], but the oxidation of methane under completely anoxic conditions has, to our knowledge, not been demonstrated experimentally for *Crenothrix* or any of the gamma-proteobacterial MOB. Nonetheless, although a plausible metabolic mechanism is still missing, they have been shown to incorporate ^13^C into their membrane lipids during biosynthesis from isotopically labeled methane in anoxic lake-sediment incubations [79].

### Water column stability as an ecological factor fostering nitrite-dependent anaerobic methane oxidation

**Fig. 6:**
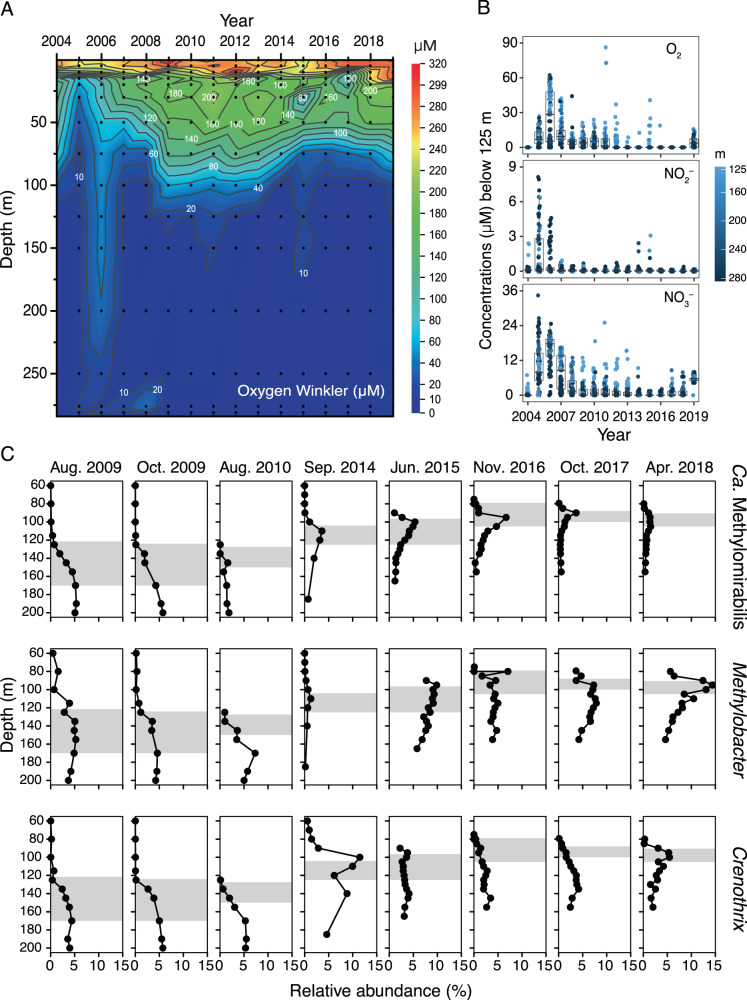
Multiannual dynamics of water column chemistry and dominant methanotrophs. **A** Lake Lugano North Basin water column oxygen concentrations data from 2005–2018 (August data shown). **B** Concentrations of potential electron acceptors for MOB introduced into water layers ≥125 m. Boxplots show median concentration values and the interquartile range. All data from a given year are shown as individual dots (*n* = 192 per year). Dot color indicates the approximate sampling depth. **C** Multiannual dynamics of *Ca*. Methylomirabilis, *Methylobacter* sp., and *Crenothrix* sp.in the water column of the North Basin of Lake Lugano, starting three years after the exceptional mixing events in 2005 and 2006. Data are based on relative read abundances of 16 S rRNA gene sequences. The extension of the redox transition zone is indicated by gray bars for the different sampling timepoints.

After nearly 50 years of meromixis in the North Basin of Lake Lugano, two exceptionally strong mixing events in 2005 and 2006 led to a complete ventilation of the water column and a massive reduction of the pelagic methane inventory from 2800 tons to 3 tons within just 1 month [50, 51]. The water column re-stabilized rapidly in the following year, and remained stratified, with anoxia below 100–125 m depth until today (Fig. 6A). As a consequence of the intrusion of oxygen to deep hypolimnetic waters, ammonium was also oxidized almost completely, leading to transiently increased nitrite and, subsequently, nitrate concentrations below 125 m depth (Fig. 6B). While we have no DNA data from the mixing period itself, in 2009 we observed unusual vertical distribution patterns and high relative abundances of three dominating groups of MOB within and below the redox transition zone: *Methylobacter* sp., *Crenothrix* sp., and *Ca*. Methylomirabilis (Fig. 6C). Water column mixing, e.g., during fall overturn in eutrophic lakes, can lead to blooms of aerobic methanotrophs in the entire water column [80]. We suggest that this also happened during the water column mixing in 2005 and 2006, and that the assemblage of MOB at still high relative abundances below the redox transition zone is partly a legacy signal from that major bloom event some years before. While in the oxic water column, such signals are readily eliminated by the regular community turnover, it may have prevailed for a longer period of time under anoxic conditions, where community turnover is slower due to reduced or absent grazing pressure by protists. Moreover, increased levels of oxygen and nitrate below 125 m until 2008 (Fig. 6B), may have helped maintaining the high relative abundances of the three MOB groups.

In 2010, in contrast to the previous years, a distinct pattern was observed for the declining *Ca*. Methylomirabilis population with respect to the other MOB (Fig. 6C). This could hint to a narrower ecological niche for *Ca*. Methylomirabilis, depending more strictly on the presence (or cryptic formation) of oxidized nitrogen compounds, which, by 2010, had been depleted below 125 m (Fig. 6B). On the other hand, the persistence of *Methylobacter* sp. and *Crenothrix* sp. also in deeper waters, could be explained by a metabolic lifestyle independent of external electron acceptors and, possibly, even of methane [75]. Indeed, mixed-acid fermentation and hydrogen production has been proposed previously based on metagenomic evidence for both *Crenothrix* D3 from lake Zug [77], as well as for *Methylobacter* sp. [46].

More stable water column conditions between 2014–2016 led to a shallowing of the redox transition zone with only few transient injections of oxidants into water depths below 125 m (Fig. 6B). During that period of time, *Ca*. Methylomirabilis was consistently peaking within the redox transition zone at 110 m, 100 m, and 95 m depth, respectively, representing up to 3.6% of total sequences in September 2014, 5.4% in June 2015, and 6.7% in November 2016. The read-based relative abundances of *Ca*. Methylomirabilis were confirmed by qPCR (Supplementary Figs. S8 and S9), showing that *Ca*. Methylomirabilis 16 S rRNA gene abundances increased from 1.5·10^4^ copies/mL in September 2014 to 3·10^6^ copies/mL in November 2016. By that time, *Ca*. Methylomirabilis had become the most abundant MOB in the redox transition zone, suggesting that water column stability was a major environmental factor promoting the growth of this slow-growing nitrite-dependent anaerobic methanotroph.

Links between water column stability and the growth of *Ca*. Methylomirabilis seem additionally confirmed by the most recent data. Enhanced mixing in 2017 and 2018 resulted in a deepening of the redox transition zone (Fig. 6A, C), and the decline and spreading of *Ca*. Methylomirabilis (Fig. 6C). While the ventilation of the upper monimolimnic water levels led to increased nitrate concentrations from stimulated nitrification (Fig. 6B), Ca. Methylomirabilis did not seem to benefit from this situation, likely because the detrimental effects of oxygenation due to deeper mixing seemed to outweigh the positive effect of the enhanced oxidant supply. Indeed, experimental studies with a *Ca*. Methylomirabilis enrichment culture have revealed that even in presence of low oxygen levels, rates of methane and nitrite conversion are strongly reduced, and cell-division-associated genes are downregulated [81]. In contrast, the more dynamic water column conditions in 2017 and 2018 stimulated the faster growing aerobic MOB, *Methylobacter* sp. but also *Crenothrix* sp., which also dominate in the dynamic South Basin water column.

While under stable water column conditions, oxygen and methane are brought together through diffusive processes, and at low concentrations, mixing events lead to transient but often strongly increased solute concentrations that affect directly the kinetics and pathways of biogeochemical processes [82]. As for methanotrophic communities, stable environmental conditions with low substrate concentrations thus are selecting for MOB with higher enzyme affinities but slower growth. Conversely, mixing-induced higher substrate levels will select for fast responding, aerobic methanotrophs. It is thus likely the interplay between stable and intermittently perturbed environmental conditions, at the redox transition zone of the meromictic North Basin, that allow the long-term coexistence of aerobic and anaerobic MOB, *Methylobacter* sp., *Crenothrix* sp, and *Ca*. Methylomirabilis.

## Conclusions

Amplicon sequencing data of 16 S rRNA genes and experimental data from anoxic ^14^CH_4_ incubations with nitrate/nitrite addition provide evidence that nitrite-dependent AOM is an important methane sink in the permanently stratified North Basin of Lake Lugano, and that this process is primarily mediated by *Ca*. Methylomirabilis at the redoxcline and in the anoxic water column. Time series data covering nearly 10 years of microbial community dynamics show that the assemblage of methanotrophs is dominated by few abundant taxa besides *Ca*. Methylomirabilis, including mainly *Methylobacter* and *Crenothrix*. These data also demonstrate that stable low-redox conditions in the midwater depths of the meromictic North Basin are particularly conducive to the development of N-AOM-performing bacteria. In the more dynamic South Basin, the assemblage of MOB consisted only of aerobic *Methylobacter* and *Crenothrix*. The duration of seasonal stratification and anoxia appears to be too short, in relation to the slow growth rate of *Ca*. Methylomirabilis, to allow for the establishement of this anaerobic methanotroph, despite favorable hydrochemical conditions. This conclusion may be extended to other lake systems, and explain why AOM usually is not a major mode of methane removal in seasonally anoxic eutrophic lakes.

Our research on methanotrophy in the two hydrodynamically differing lake basins highlights that the stability of environmental/redox conditions, on the one hand, and physical processes, like mixing and water column dynamics, on the other, are important factors determining microbial community structures as well as pathways and rates of biogeochemical processes. With climate warming, lake surface temperatures will continue to increase, leading to enhanced density stratification and reduced vertical mixing. In particular, deeper lakes may transition from a monomictic to an oligo- or meromictic mixing regime [83], which means that the mitigating effects of anaerobic methane oxidation on methane emissions from freshwater systems will probably increase in the future.

## Supplementary information

Supplementary Material

S1

## Data Availability

Raw sequence data are made available at NCBI under the BioProjectID PRJNA672280 with the accession numbers SRR12936362 through SRR12936382; MH111698 through MH113143. Treated 16 S rRNA gene sequences (ASV) and water column chemistry data used for figures are deposited in the Zenodo repository (10.5281/zenodo.7621528).
