# Supplementary material for: Water column dynamics control nitrite-dependent anaerobic methane oxidation by Candidatus “Methylomirabilis” in stratified lake basins
Source: ISME J. 2023 Feb 20;17(5):693–702. doi: 10.1038/s41396-023-01382-4 (PMC10119105; doi:10.1038/s41396-023-01382-4)
Supplement: Supplementary file 1 — Supplementary Material [file 41396_2023_1382_MOESM1_ESM.pdf]

Supporting Information

Water column dynamics control nitrite-dependent anaerobic oxidation of methane by *Candidatus* 'Methylomirabilis' in stratified lake basins

Guangyi Su[1, 2], Moritz F. Lehmann[1], Jana Tischer[1], Yuki Weber[1], Fabio Lepori[3], Jean-Claude Walser[4], Helge Niemann[1, 5], Jakob Zopfi[1]

[1] Department of Environmental Sciences, University of Basel, Basel, Switzerland

[2] State Key Laboratory of Marine Environmental Science, College of Ocean and Earth Sciences, Xiamen University, Xiamen, China.

[3] Department for Environment, Constructions and Design, University of Applied Sciences and Arts of Southern Switzerland (SUPSI), Mendrisio, Switzerland

[4] Genetic Diversity Centre (GDC), ETH Zürich, Zürich, Switzerland

[5] Department of Marine Microbiology and Biogeochemistry, NIOZ Royal Institute for Sea Research and Utrecht University, Texel, The Netherlands

Corresponding authors: guangyisu@xmu.edu.cn and jakob.zopfi@unibas.ch

Supplementary Methods

Preparation of 16S rRNA gene amplicon library

The amplification conditions of the initial PCR during library generation are critical, and can significantly affect results. Too few amplification cycles will limit the detection of low abundant taxa, whereas too many cycles may lead to PCR product saturation and to a biased representation of microbial community structures. In order to optimize our library preparation protocol, we evaluated the effect of two different amplification cycle numbers (18 vs. 25 cycles) for the initial PCR step, resulting in two libraries, which were sequenced in the same Illumina run.

The initial PCR consisted of 12.5 μL of 2X KAPA HiFi Hot Start Ready Mix, 0.75 μL forward primer (10 μM), 0.75 μL reverse primer (10 μM), 1 μL of PCR grade water, and 10 μL of template DNA (1 ng μL^{-1}). Four sets of 16S rRNA primers (Table S1) were used to amplify sample DNA. The primers contained 0-3 additional ambiguous bases between the adapter sequence and the PCR primer 515F-Y/926R [1] to increase the nucleotide diversity and to improve template generation during Illumina sequencing. Increasing the nucleotide diversity at this stage allowed using a relatively low amount of PhiX (10 %) during sequencing. The primer concentration used in our assay corresponded to 1.5x the concentration described in the Illumina 16S metagenomic library preparation guide (https://support.illumina.com/documents/documentation/chemistry_documentation/16s/16s-metagenomic-library-prep-guide-15044223-b.pdf). PCR was run on a Biometra thermocycler using the following program: 98 ºC for 3 min; cycles of 98 ºC for 20 seconds, 55 ºC for 15 seconds, and 72 ºC for 15 seconds (18 or 25 cycles, respectively); 72 ºC for 5 minutes final elongation. Samples were then cleaned using AMPure Beads following the manufacturer's instructions. Nextera XT index primers (N7XX and S5XX; Illumina) were attached to the amplicons in a subsequent 25 μL PCR reaction with: 12.5 μL of 2X KAPA HiFi, 2.5 μL each of Nextera XT index primer 1 and 2, 2.5 μL of PCR water and 5 μL of the cleaned amplicon run at 95 ºC for 3 min; 8 cycles of 95 ºC for 30 seconds, 55 ºC for 30 seconds, and 72 ºC for 30 seconds; 72 ºC for 5 minutes. Products were again cleaned using AMPure Beads and quality-checked using Agilent Fragment analyzer (dsDNA-915 reagent kit), quantified using Qubit (ThermoFisher), normalized and finally pooled at equimolar quantities. Sequencing was done on a MiSeq system (Illumina), using the

PE 300 method (V3 reagent kit). Library preparation and sequencing was done at the Genomics Facility Basel (D-BSSE ETHZ and Basel University). Raw sequence data are made available at NCBI under the BioProjectID PRJNA672280 with the accession numbers SRR12936362 through SRR12936382; MH111698 through MH113143.

Sequence analyses

Quality control of raw reads, initial sequence treatment, and taxonomic assignment is described in the main manuscript. Sequencing results are summarized in Table S2. 16S rRNA sequence data were analyzed in R (v3.5.1) (R Core Team, 2014, http://www.r-project.org/) using mostly the libraries: phyloseq [2], vegan [3], and ggplot2 [4]. Rarefaction curves show that 25 cycles yielded better estimates of the species richness than 18 cycles (Figure S1). ANOVA in R was used to test whether different alpha diversity measures (i.e., Observed richness, Chao1, Shannon and InvSimpson) were affected by either the sequencing depth or the amplification cycle number. Results show (Table S3) that Shannon is significantly affected by both the amplification cycle number and sampling depth, while Chao1 depends on the sampling depth [5]. Accordingly, samples were rarefied for alpha diversity estimates and for comparing the numbers amplified sequence variants (ASVs) in the two libraries (Table S2). Principal coordinate analysis (PCoA) shows that both 18 and 25 PCR amplification cycles yielded very similar microbial community structures (Figure S3). Ultimately we choose 25 amplification cycles for the microbial community analysis, because of the better sensitivity towards minor taxa. Sample Ga_100m in the 25 cycle library had particularly low read and ASV numbers (Table S2) and was excluded from any further analysis. After removing sample Ga_100m and eliminating both mitochondrial and chloroplast 16S rRNA gene reads, as well as empty ASVs, a total of 8717 ASVs remained in the unrarefied 25-cycle library dataset. No further filtering of low abundant reads was done.

In this dataset we identified 41 ASVs of potential methanotrophs (Supplementary Excel File S1) in the two basins of Lake Lugano, North Basin and South Basin. The guild of methanotrophs was dominated by seven highly abundant ASVs with relative abundances of > 1 % in at least one sample, including sequences of uncultured *Methylobacter* sp. (ASV19, ASV42, ASV5, ASV18), uncultured *Crenothrix* sp. (ASV10, ASV91), as well as *Ca.* Methylomirabilis (ASV9). Four ASVs in both the North Basin and South Basin were of intermediate abundance (< 1-0.1 % relative

abundance), but most of the potential methanotroph ASVs (21 in North Basin; 23 in South Basin) were low-abundant with < 0.1 % (Supplementary Excel File S1).

Of the 41 ASVs of potential methanotrophs, 20 ASVs were common in both lake basins, whereas 9 ASVs were only detected in the North Basin and another 12 in the South Basin only. The two latter groups consisted of low-abundant taxa, thus the apparent habitat specificity was rather a consequence of incomplete community sampling by sequencing than reflecting true ecological preference for one or the other basin. The fact that the 16S rRNA gene sequences of the MOB common to both basins are identical suggests that the two basins are microbiologically connected.

Clear habitat preference, however, was observed for the highly abundant ASV9 (*Ca.* Methylomirabilis), which was consistently detected in the water column of the meromictic North Basin of Lake Lugano only. In the South Basin, *Ca.* Methylomirabilis was not detectable in any water-column samples. Nonetheless, ASV9 was present in a surface sediment (0-2 cm depth) sample retrieved at 93 m depth, suggesting that sedimentary *Ca.* Methylomirabilis could colonize the anoxic bottom water for the South Basin, provided that the environmental conditions were favorable.

Supplementary tables

Table S1. Design of the four different sets of forward and reverse primers used for library preparation. The primers contained 0-3 additional ambiguous bases (indicated in bold red) between the adapter sequence and the amplicon PCR primer 515F-Y/926R [1] to increase the nucleotide diversity and improve template generation during Illumina sequencing.

<table>
<tr><th colspan="2">Primer</th><th>Sequence</th></tr>
<tr><td colspan="2">515F-Y</td><td>GTGYCAGCMGCCGCGGTAA</td></tr>
<tr><td colspan="2">926R</td><td>CCGYCAATTYMTTTRAGTTT</td></tr>
<tr><td rowspan="4">Forward primer sets</td><td>16S_Par0_fw</td><td>5’- TCGTCGGCAGCGTCAGATGTGTATAAGAGACAG-GA-GTGYCAGCMGCCGCGGTAA-3’</td></tr>
<tr><td>16S_Par1_fw</td><td>5’- TCGTCGGCAGCGTCAGATGTGTATAAGAGACAG-**N**-GA-GTGYCAGCMGCCGCGGTAA -3’</td></tr>
<tr><td>16S_Par2_fw</td><td>5’- TCGTCGGCAGCGTCAGATGTGTATAAGAGACAG-**NN**-GA-GTGYCAGCMGCCGCGGTAA -3’</td></tr>
<tr><td>16S_Par3_fw</td><td>5’- TCGTCGGCAGCGTCAGATGTGTATAAGAGACAG-**NNN**-GA-GTGYCAGCMGCCGCGGTAA -3’</td></tr>
<tr><td rowspan="4">Reverse primer sets</td><td>16S_Par0_rev</td><td>5’- GTCTCGTGGGCTCGGAGATGTGTATAAGAGACAG-CA-CCGYCAATTYMTTTRAGTTT -3</td></tr>
<tr><td>16S_Par1_rev</td><td>5’- GTCTCGTGGGCTCGGAGATGTGTATAAGAGACAG-**N**-CA-CCGYCAATTYMTTTRAGTTT -3’</td></tr>
<tr><td>16S_Par2_rev</td><td>5’- GTCTCGTGGGCTCGGAGATGTGTATAAGAGACAG-**NN**-CA-CCGYCAATTYMTTTRAGTTT-3’</td></tr>
<tr><td>16S_Par3_rev</td><td>5’- GTCTCGTGGGCTCGGAGATGTGTATAAGAGACAG-**NNN**-CA-CCGYCAATTYMTTTRAGTTT-3’</td></tr>
</table>

Table S2. Comparison of the Lake Lugano 16S rRNA gene sequence data of two libraries prepared from the same original DNA, but with 18 and 25 amplification cycles, respectively. The two libraries were sequenced together in a single Illumina run. Total reads per sample after sequencing are given for the two libraries, as well as the number of amplified sequence variants (ASVs). Selected alpha diversity measures were determined after rarefying to even sequencing depths.

Samples \ Cycles	Reads		ASVs		Alpha diversity: Chao1		Alpha diversity: Shannon		Alpha diversity: InvSimpson	
	**18**	**25**	**18**	**25**	**18**	**25**	**18**	**25**	**18**	**25**
**Fi_65m**	38540	98913	1120	1429	735.7	865.9	4	4	18	16.3
**Fi_70m**	34898	33335	1029	1141	706.4	810.5	3.9	4.4	15.1	25
**Fi_75m**	29012	29961	1012	1144	736.3	1067.2	4.4	4.4	30.2	35.3
**Fi_80m**	27959	32000	1100	1349	939.5	1044.7	4.3	4.6	24.1	32.3
**Fi_85m**	52912	38948	1470	1454	906.3	1057.5	4.1	4.4	14.8	20.4
**Fi_90m**	57085	68013	1780	2300	1113	1264.9	4.4	4.7	21.2	31.9
**Ga_75m**	31248	34349	866	1087	759.6	921.8	3.5	4.2	5.4	11.6
**Ga_80m**	39354	111063	923	1322	784.7	888.5	3.4	3.3	4.9	4.8
**Ga_85m**	32757	105780	1109	1767	784.8	1104.7	4.5	4.2	30.6	16
**Ga_90m**	31711	45194	1113	1367	936	1035.5	4.2	4.4	18.3	21.2
**Ga_95m**	24019	45002	1052	1471	872.2	1163.7	4.2	4.3	19.3	24.1
**Ga_100m**	15107	**6781**	822	**77**	815.4	**75**	3.7	3.5	7.5	23.9
**Ga_105m**	16999	57092	882	1672	889.6	973.1	3.9	4.2	11.1	18.8
**Ga_110m**	16531	43350	968	1635	831.7	1010.8	4.2	4.3	14.4	18.8
**Ga_115m**	15152	40296	931	1690	818.1	1192.4	4	4.3	9.8	14.2
**Ga_120m**	27566	62274	1334	2158	978	1152.8	4.1	4.5	11.1	18.3
**Ga_125m**	37849	39652	1771	1924	1262	1331.9	4.3	4.5	12.2	18.5
**Ga_130m**	60858	35336	2036	1736	1296.5	1208.9	4.1	4.6	9.6	17
**Ga_135m**	37645	36491	1811	1885	1225.1	1417.6	4.2	4.6	9.3	17.9
**Ga_145m**	29216	38769	1450	1809	1087.2	1403.9	3.8	4.1	6.2	9
**Ga_155m**	25340	40981	1461	2001	1203	1300.8	4	4.2	7.5	10.3
**Mean**	32465	49694	1240	1544	937	1062	4	4	14	19
**Total**	681758	1043580	26040	32418	–	–	–	–	–	–

Table S3. Results of a two-way factorial ANOVA for the effects of sequencing depth and amplification cycle number on different alpha diversity measures (Observed ASV richness, Chao1, Shannon, and Inversed Simpson). The sequencing depth (i.e. read number per sample) had a significant effect (*) on the Chao1 richness estimator and Shannon diversity, whereas the PCR amplification cycle number used for library preparation significantly affected Shannon diversity.

Indices / Factors	Observed	Chao1	Shannon	InvSimpson
Sequencing depth	F = 3.331<br>p =0.076	F = 8.171<br>p =0.007 **	F= 7.415<br>p = 0.009**	F= 3.387<br>p = 0.074
Cycle number	F = 2.030<br>p = 0.163	F = 0.137<br>p = 0.714	F= 6.175<br>p = 0.017 *	F = 1.697<br>p = 0.201

Table S4. (**A**) Results from a three-way factorial ANOVA for the effects of site (North Basin/South Basin), treatment (i.e., control/sulfate/molybdate/nitrate/nitrite), and incubation time (16 days/32 days) on the AOM rates in the incubation experiments. (**B**) *p* values from one-way ANOVA with Tukey's honest significant difference (HSD) test on different treatments in the two basins compared to the control. For both North Basin and South Basin, AOM rates from incubations of 16 and 32 days (n = 6) are pooled to see the overall difference of experimental treatments to the control. The significance level α was set at 0.05.

A

Effect	Df	F	*p* value	Significance
Site	1	62.74	1.01E-05	***
Treatment	4	8.03	7.53E-09	***
Time	1	22.08	3.08E-05	***
Treatment:Site	4	5.58	1.14E-03	**
Treatment:Time	4	1.14	0.35	
Site:Time	1	12.40	1.09E-03	**
Treatment:Site:Time	4	0.48	0.75	

B

p value	Difference to control			
	Molybdate	Sulfate	Nitrate	Nitrite
North Basin	0.58	0.99	**< 0.01**	**0.05**
South Basin	0.84	0.95	0.68	0.98

Supplementary figures

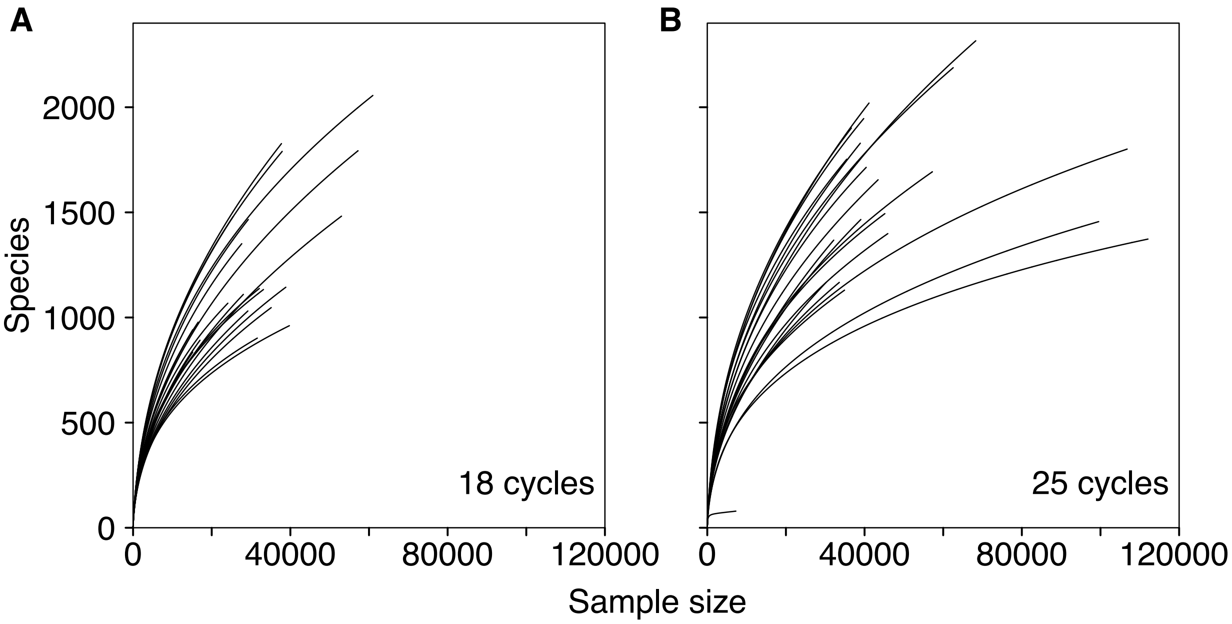

Figure S1. Rarefaction curves of samples from libraries generated with different PCR amplification cycles: (A) 18 cycles and (B) 25 cycles. Each curve represents one sample, showing the cumulative number of new ASVs ("Species") in a given number of sampled sequences ("Sample size").

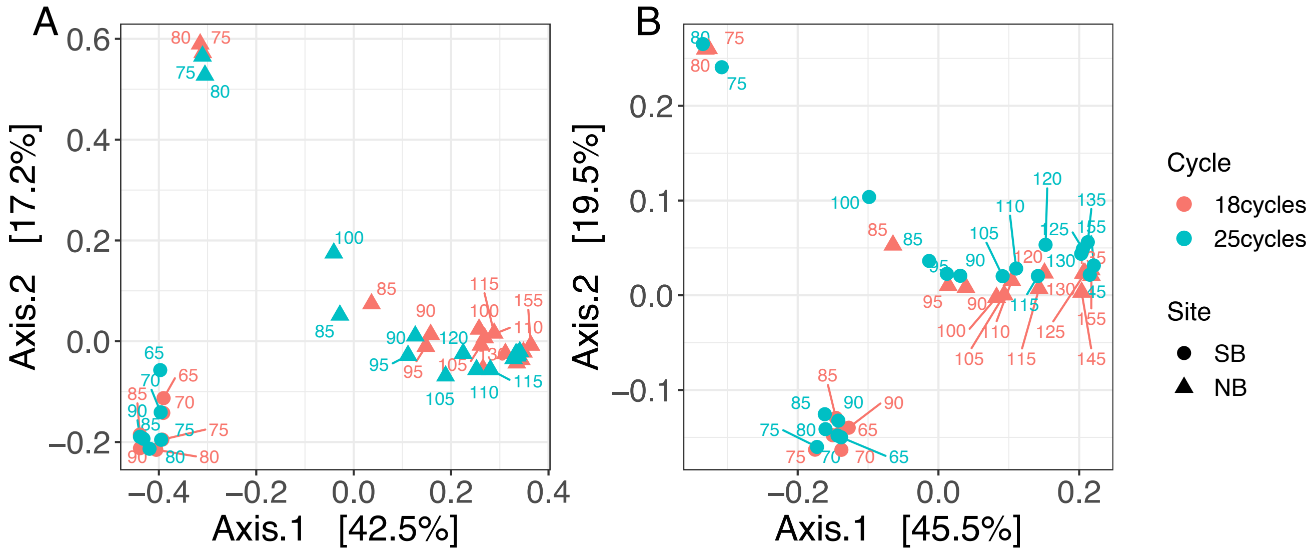

Figure S2. Principal coordinate analysis (PCoA) of microbial community structures in samples of the North Basin (NB) and South Basin (SB) in Lake Lugano with two different PCR amplification cycle numbers (18 cycles and 25 cycles). Plots of PCoA with (A) Bray-Curtis and (B) weighted UniFrac distances revealed an atypical community structure for one of the samples (Ga_100 m, 25 cycles, filled cyan circle, 100), possibly caused by a PCR amplification artifact. The x- and y-axes represent the first and second coordinates, respectively, and the values in square brackets show the percentages of the community variation explained.

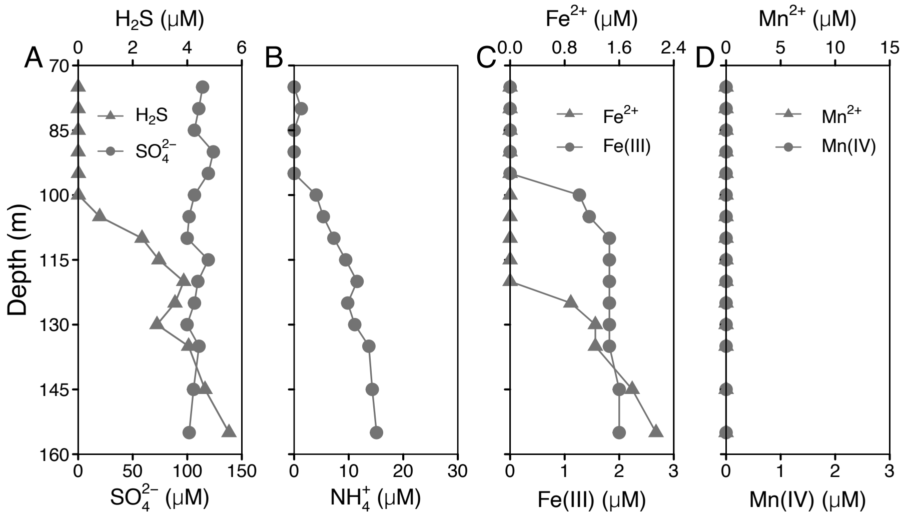

Figure S3. Concentration profiles of (A) sulfate and sulfide, (B) ammonium, (C) particulate Fe(III)-oxides and dissolved (Fe^{2+}) iron species, and (D) manganese species in the mid water column of the permanently stratified North Basin of Lake Lugano in November 2016. Both dissolved Mn^{2+} and particulate Mn(IV)-oxide concentrations were below detection limit throughout the investigated depths.

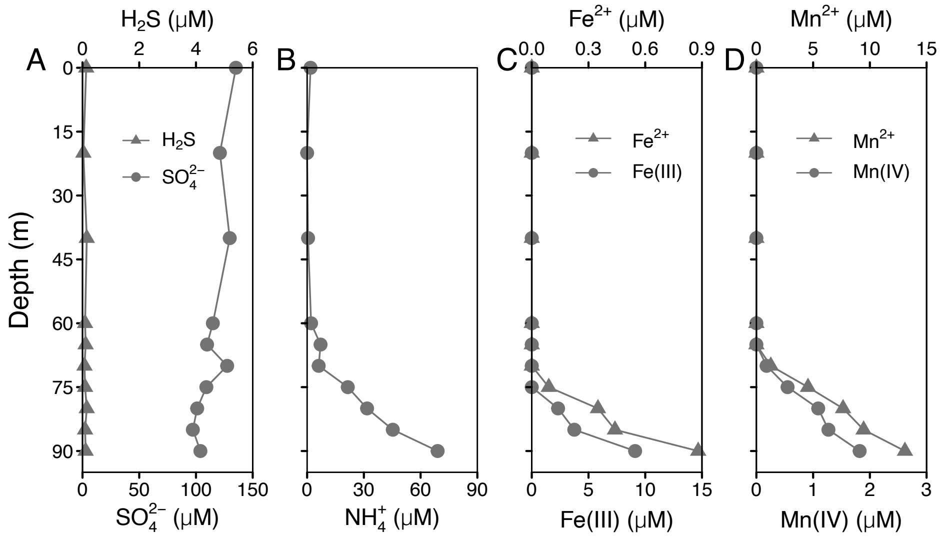

Figure S4. Concentration profiles of (A) sulfate and sulfide, (B) ammonium, (C) particulate Fe(III)-oxides and dissolved (Fe^{2+}) iron species, and (D) Mn(IV)-oxides and dissolved (Mn^{2+}) manganese in the water column of the seasonally stratified South Basin of Lake Lugano in November 2016.

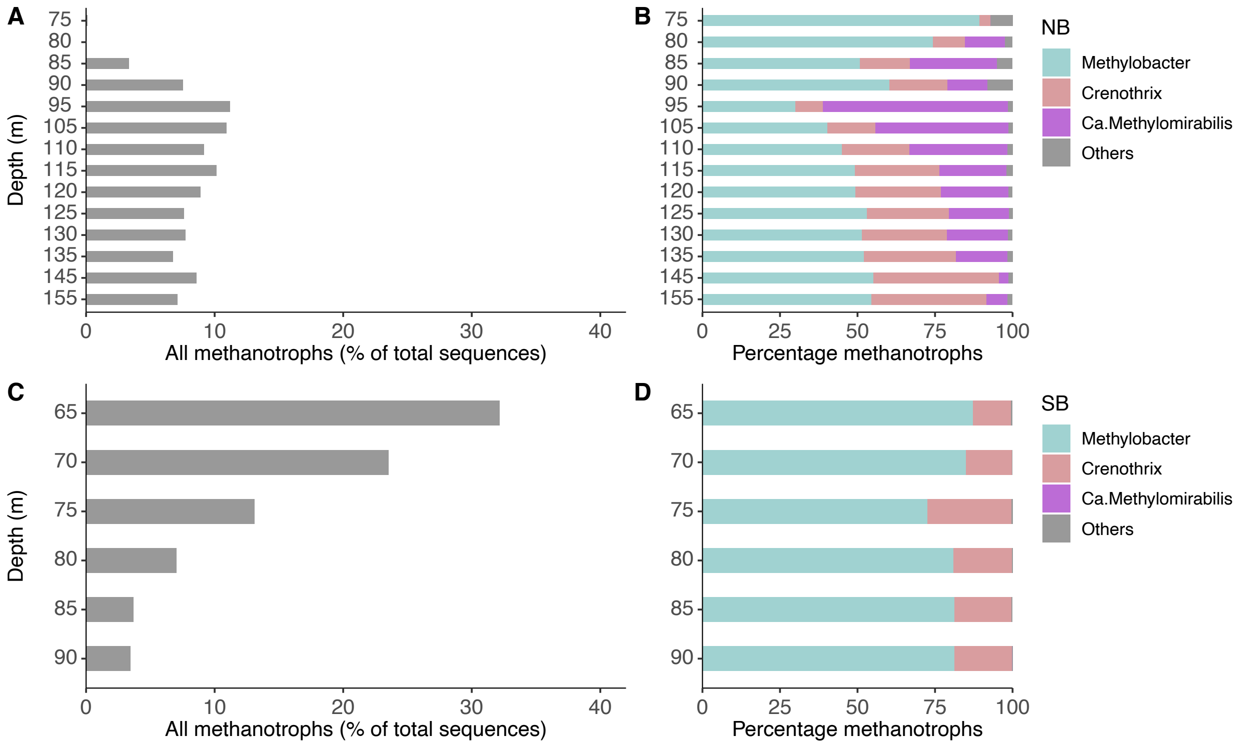

Figure S5. Depth profiles of summed relative abundances of methanotrophs in the entire community in the North Basin (NB) and South Basin (SB), respectively (A and C). The percentage of the dominant methanotrophs within the methanotroph assemblage at different depths is shown in B and D: *Methylobacter* sp. (ASV5, ASV18, ASV19, ASV42; See Supplementary Excel File S1), *Crenothrix* sp. (ASV10, ASV91), and *Candidatus* Methylomirabilis (ASV9). Data are from Lake Lugano water column of NB (A and B) and SB (C and D) in November 2016. *Ca. Methylomirabilis* was not detected in the SB water column. Data are based on relative read abundances of 16S rRNA gene amplicons.

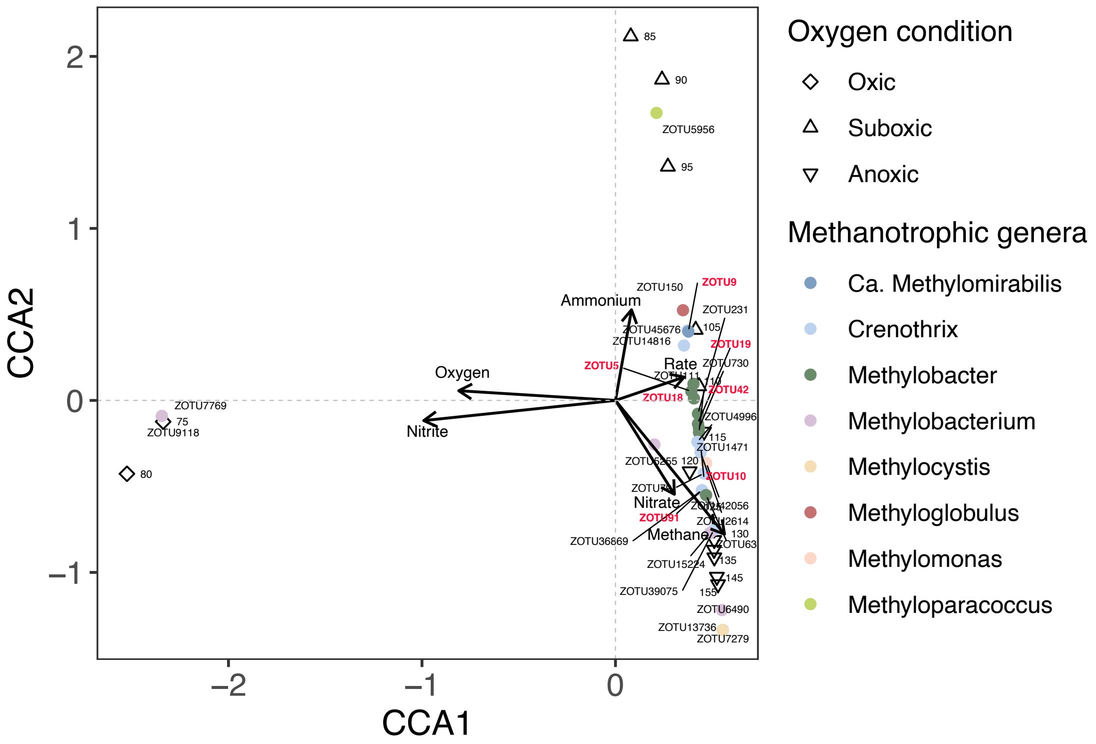

Figure S6. Canonical correspondence analysis (CCA) based on nutrient concentrations, AOM rate measurements, and potential methanotrophs in the North Basin of Lake Lugano. Open triangles and diamonds represent samples in this basin under different oxygen conditions (Oxic: > 5 µM, Anoxic: < 0.5 µM), with numbers indicating the water depths. Ordination was performed using 'vegan' in R. Taxa abundance and environmental variables (concentrations and rates) were Hellinger transformed prior to ordination. The CCA triplot shows that *Ca.* Methylomirabilis (ZOTU = ASV9, blue filled circle) is found in the suboxic (O_2 < 5 µM) water column of the permanently stratified North Basin (open triangles, e.g., at 105 m), and is positively related to the methane oxidation rate. The plot also shows that *Ca.* Methylomirabilis (ASV9, in bold red), *Methylobacter* sp. (ASV19, ASV42, ASV5, ASV18, in bold red), and *Crenothrix* sp. (ASV10, ASV91, in bold red) are anti-correlated to nitrite concentrations, indicating that they may be responsible for the depletion of the nitrite pool in the habitat. Arrows represent solute concentrations, with arrowheads indicating their direction of increase.

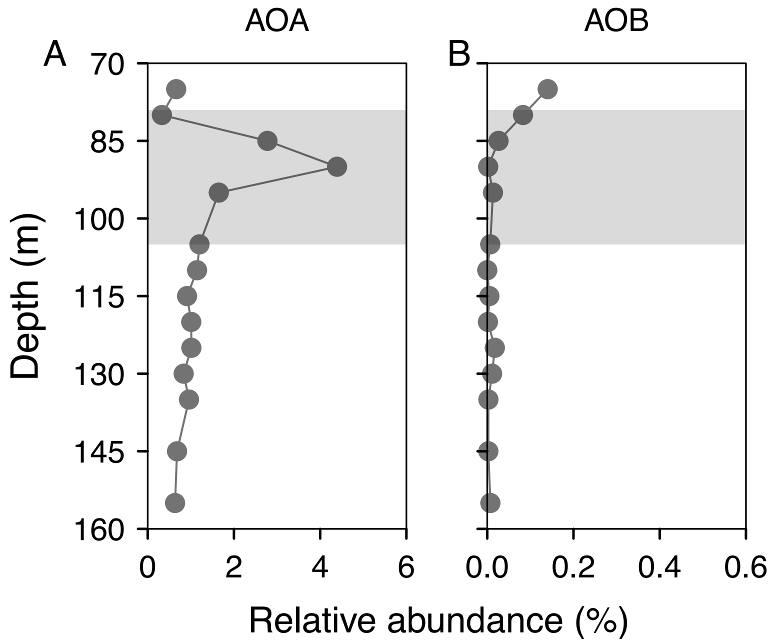

Figure S7. Depth profiles of relative abundances of (A) ammonium-oxidizing archaea (AOA, *Ca.* Nitrosopumilus), and (B) ammonium-oxidizing bacteria (AOB, *Nitrosomonas* and *Nitrosospira*) in the North Basin water column of Lake Lugano in November 2016. The grey area represents the redox transition zone. Data are based on relative read abundances of 16S rRNA gene amplicons.

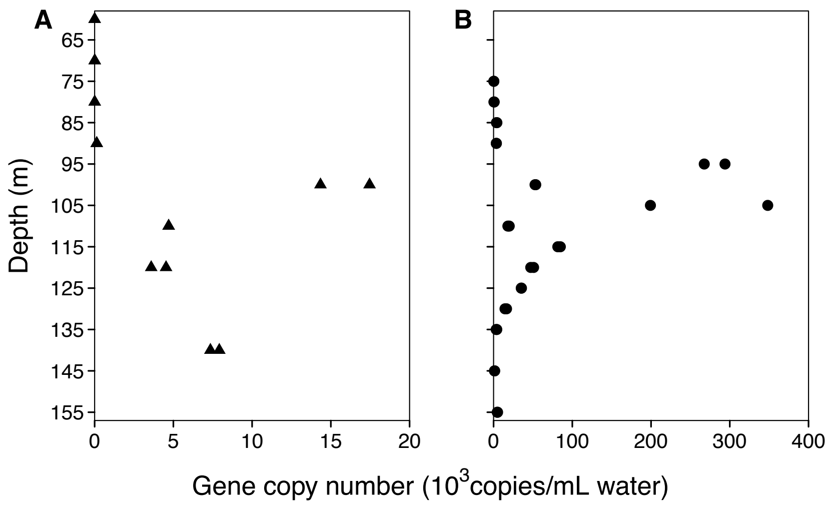

Figure S8. Depth profiles of 16S rRNA gene copy numbers of *Ca.* Methylomirabilis in the water column of the permanently stratified North Basin of Lake Lugano in (A) September 2014 and (B) November 2016. The redoxcline was located between 104 and 125 m in 2014, and between 79 and 105 m in 2016. Based on qPCR data (i.e., the local maximum gene copy numbers between the two years), the apparent doubling time of *Ca.* Methylomirabilis in the lake water column was approximately 104 days.

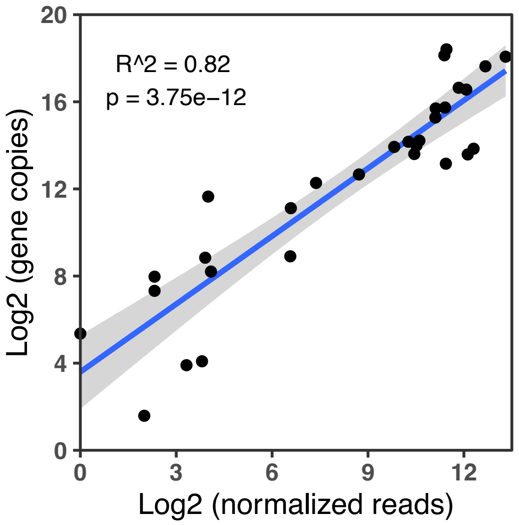

Figure S9. Relationship between 16S rRNA gene copy numbers and the normalized (to equal sequencing depth) reads of *Ca.* Methylomirabilis in the North Basin water column. Samples are from October 2010, September 2014 and November 2016.

References

1. Parada AE, Needham DM, Fuhrman JA. Every base matters: Assessing small subunit rRNA primers for marine microbiomes with mock communities, time series and global field samples. *Environ Microbiol* 2016; **18**: 1403–1414.
2. McMurdie PJ, Holmes S. Phyloseq: an R package for reproducible interactive analysis and graphics of microbiome census data. *PLoS One* 2013; **8**: 1–11.
3. Oksanen J, Blanchet FG, Kindt R, Legendre P, Minchin P, O'Hara R, et al. Vegan: community ecology package. *R Package version* 2013.
4. Wickham H. ggplot2: elegant graphics for data analysis. 2009. Springer, New York, NY.
5. Weiss S, Xu ZZ, Peddada S, Amir A, Bittinger K, Gonzalez A, et al. Normalization and microbial differential abundance strategies depend upon data characteristics. *Microbiome* 2017; **5**: 27.
